# Fluid biomarkers for neurodegenerative diseases: a comprehensive update

**DOI:** 10.1186/s13195-025-01919-z

**Published:** 2025-12-20

**Authors:** Martina Valletta, Nils Briel, Idil Yuksekel, Michelle Barboure, Anna Coward, Julie F. H. De Houwer, Ayesha Fawad, Alberto González-Mayoral, Gianmarco Iaccarino, Francisco Martínez-Dubarbie, Shirine Moukaled, Ulf Andreasson, Johan Gobom, Ann Brinkmalm, Betty Tijms, Henrik Zetterberg, Kaj Blennow, Marc Suárez-Calvet, Michael Schöll, Ross W. Paterson, Laia Montoliu-Gaya, Aitana Sogorb-Esteve

**Affiliations:** 1 Aging Research Center, Department of Neurobiology, Care Sciences and Society, Karolinska Institutet and Stockholm University, Stockholm, Sweden; 2 Department of Neurology, University Hospital Zurich, Zurich, Switzerland; 3 Zurich Neuroscience Center, University of Zurich, Zurich, Switzerland; 4 Paris Brain Institute, Sorbonne Université, INSERM U1127, CNRS 7225, Hôpital Pitié-Salpêtrière, Paris, France; 5 Alzheimer Center Amsterdam, Neurology, Vrije Universiteit Amsterdam, Amsterdam UMC Location VUmc, Amsterdam, the Netherlands; 6 Amsterdam Neuroscience, Neurodegeneration, Amsterdam, the Netherlands; 7 Barcelona Beta Brain Research Center (BBRC), Pasqual Maragall Foundation, Barcelona, Spain; 8 Centro de Investigación, Biomédica En Red - Fragilidad y Envejecimiento Saludable (CIBER-FES), Madrid, Spain; 9 Universitat Autònoma de Barcelona, Barcelona, Spain; 10 Department of Neurology and Alzheimer Centre, Erasmus MC University Medical Centre, Rotterdam, the Netherlands; 11 Clinical Memory Research Unit, Department of Clinical Sciences, Lund University, Malmo, Sweden; 12 Hospital del Mar Research Institute, C. del Dr. Aiguader 88, Barcelona, Spain; 13 Neurology Service, Marqués de Valdecilla University Hospital, Santander, Cantabria Spain; 14 Institute for Research Marqués de Valdecilla (IDIVAL), Santander, Cantabria Spain; 15 Network Center for Biomedical Research in Neurodegenerative Diseases, CIBERNED, National Institute of Health Carlos III, Madrid, Spain; 16 Department of Epidemiology, School of Public Health and Tropical Medicine, Tulane University, New Orleans, LA USA; 17 Department of Psychiatry and Neurochemistry, Institute of Neuroscience and Physiology, the Sahlgrenska Academy at the University of Gothenburg, Mölndal, Sweden; 18 Clinical Neurochemistry Laboratory, Sahlgrenska University Hospital, Mölndal, Sweden; 19 Department of Neurodegenerative Disease, Queen Square Institute of Neurology, University College London, Queen Square, London, WC1N 3BG UK; 20 UK Dementia Research Institute, University College London, Unit 3a, 338 Euston Rd.,, London, NW1 3BT UK; 21 Hong Kong Center for Neurodegenerative Diseases, Units 1501-1502, 1512-1518, 15/F Building 17 W, 17 Science Park W Ave, Science Park, Hong Kong, China; 22 UW Department of Medicine, School of Medicine and Public Health, 750 Highland Ave, Madison, WI 53726 USA; 23 Paris Brain Institute, ICM, Pitié-Salpêtrière Hospital, Sorbonne University, Paris, France; 24 Neurodegenerative Disorder Research Center, Division of Life Sciences and Medicine, and Department of Neurology, Institute on Aging and Brain Disorders, University of Science and Technology of China and First Affiliated Hospital of USTC, Hefei, People’s Republic of China; 25 Servei de Neurologia, Hospital del Mar, Passeig Marítim 25-29, Barcelona, Spain; 26 Wallenberg Centre for Molecular and Translational Medicine, University of Gothenburg, Gothenburg, Sweden; 27 Dementia Research Centre, Queen Square Institute of Neurology, University College London, London, UK; 28 Department of Psychiatry, Cognition and Aging Psychiatry, Sahlgrenska University Hospital, Mölndal, Sweden; 29 UK Dementia Research Institute at University College London, London, WC1N 3BG UK

**Keywords:** Neurodegeneration, Alzheimer's Disease, Blood-Based Biomarkers, Fluid Biomarkers, CSF Biomarkers, Neurofilament Light Chain, Glial Fibrillary Acidic Protein, Tau, Synuclein, TDP-43

## Abstract

Fluid biomarkers are revolutionizing the diagnosis and management of neurodegenerative diseases by enabling earlier diagnosis and disease monitoring. In particular, blood-based biomarkers have emerged as a minimally invasive and scalable alternative to cerebrospinal fluid analysis. Recent advances in blood-based tau biomarkers have shown high diagnostic accuracy for Alzheimer’s disease (AD). Other neurodegenerative diseases—such as synucleinopathies, frontotemporal lobar degeneration, limbic-predominant age-related TDP-43 encephalopathy (LATE), and amyotrophic lateral sclerosis—pose substantial challenges due to their heterogeneous clinical presentations and the current absence of robust biomarkers for hallmark pathologies. Nonetheless, promising candidate markers are emerging for improved disease characterization and staging. Technological innovations, including single-molecule arrays (Simoa), advanced mass spectrometry workflows and nucleic acid linked immune-sandwich assay (NULISA) have markedly enhanced the sensitivity and precision of biomarker quantification from low-concentration biological matrices. More recently, the development of fully automated platforms shows great promise for routine measurement of blood-based biomarkers in clinical settings. Despite this progress key challenges remain, including the need for improved assay reproducibility, standardization, and the optimization of clinical workflows. In this review, we provide a comprehensive update on recent progress in fluid biomarker research across AD and major neurodegenerative diseases, highlight technological advances in detection methods, and discuss current challenges and opportunities for clinical translation.

**Supplementary Information:**

The online version contains supplementary material available at 10.1186/s13195-025-01919-z.

## Background

Fluid biomarkers are valuable tools for the evaluation of patients with neurodegenerative diseases [1]. Cerebrospinal fluid (CSF) biomarkers have been extensively studied in research settings and used in clinical routine for selected diseases. More recently, blood-based biomarkers (BBMs) have emerged, offering a less invasive and more accessible method for detecting neuropathological changes [2]. Beyond their diagnostic value, quantification of biomarkers in easily accessible biological fluids holds promise for monitoring disease progression over time. In the context of clinical trials, fluid biomarkers are increasingly applied for patient stratification, enabling the enrolment of biologically defined subgroups, and could potentially support the evaluation of therapeutic efficacy.

Notable progress has been made in the development of fluid biomarkers for Alzheimer’s disease (AD), the most prevalent neurodegenerative disease and the leading cause of dementia (Table 1) [3]. The National Institute on Aging and Alzheimer's Association research guidelines proposed a significant shift towards a biological definition of AD, based on biomarkers of amyloid pathology (A), tau (T) and neurodegeneration (N) [4]. This framework was revised and updated in the 2024 Alzheimer's Association guidelines for Alzheimer´s disease diagnosis and staging [5], according to which abnormalities of any “core 1” biomarker – such as markers of amyloid-β (Aβ) proteinopathy or soluble phosphorylated-tau (p-tau) – are sufficient to establish a biological diagnosis of AD, regardless of the presence of clinical symptoms. Alternative frameworks have also been proposed, such as that of the International Working Group, which proposes AD as a clinical-biological construct and emphasizes the integration of biomarkers with clinical features [6].

**Table 1:** Overview of neurodegenerative diseases covered in the review

Entity	Clinical Picture	Hallmark Pathology	Established Diagnostic Biomarkers
Alzheimer Disease (AD)	Cognition: episodic memory, visuospatial and frontal dysexecutive impairment, aphasia Psychiatric symptoms	Extracellular plaques: Aβ aggregates Neurofibrillary tangles: hyperphosphorylated 3R/4R tau	MRI: mesial temporal atrophy PET: amyloid, fluorodeoxyglucose CSF: p-tau181/Aβ42, t-tau/Aβ42, Aβ42/40 Plasma: p-tau217, p-tau181, p-tau231
Parkinson Disease (PD)	Motor: bradykinesia, rigidity, resting tremor, postural and gait impairment Non-motor: hyposmia, autonomic dysfunction, cognitive impairment, sleep alterations	α-synuclein inclusions in neurons (Lewy bodies, Lewy neurites)	SPECT/PET: low dopamine transporter uptake in the basal ganglia Polysomnography: RBD
Dementia with Lewy Bodies (DLB)	Cognition: attention, cognitive fluctuations Visual hallucinations Parkinsonism Sleep alterations	α-synuclein inclusions in neurons (Lewy bodies, Lewy neurites) Frequent AD co-pathology	SPECT/PET: low dopamine transporter uptake in basal ganglia 123I-MIBG scintigraphy: low myocardial uptake Polysomnography: RBD
Multiple System Atrophy (MSA)	Autonomic dysfunction Parkinsonism: MSA-P Cerebellar syndrome: MSA-C Sleep alterations	α-synuclein inclusions in oligodendrocytes	MRI: “hot cross-bun” sign, atrophy of putamen, middle cerebellar peduncle, pons, cerebellum Polysomnography: RBD, stridor
Frontotemporal Lobar Degeneration (FTLD)	Cognition: behavioral and social interaction difficulties, executive dysfunction, aphasia (FTD) Motor symptoms: parkinsonism, motor neuron signs Psychiatric symptoms	FTLD-tau: cytoplasmic inclusions of 3R and/or 4R tau in neurons and glia FTLD-TDP: TDP-43 inclusions in neurons (cytoplasmic ± nuclear) FTLD-FET	MRI: frontotemporal atrophy FDG-PET: hypometabolism Genetics: NGS, repeat-primed PCR
Limbic Predominant Age-related TDP-43 Encephalopathy (LATE)	Cognition, episodic memory impairment (often isolated) Mostly > 80 years, < 80 years understudied	TDP-43 neuronal cytoplasmic aggregates in amygdala, hippocampus, middle frontal gyrus Often as co-pathology with AD neuropathology (50–70%)	MRI: mesial temporal atrophy
Amyotrophic Lateral Sclerosis (ALS)	Motor: upper and/or lower motor neuron signs; spastic and/or flaccid paresis of voluntary muscles Respiratory paralysis Cognition: FTD	TDP-43 neuronal cytoplasmic aggregates (97%) SOD1 neuronal (glial) aggregates FUS neuronal (glial) aggregates *C9orf72* repeat expansion > 30, RNA toxicity	Electrophysiology Genetics: NGS, repeat-primed PCR

*Abbreviations: 3R/4R *3-repeat/4-repeat tau*, Aβ *amyloid-beta,* ALS *amyotrophic lateral sclerosis, *CSF *cerebrospinal fluid,* DLB *dementia with Lewy bodies,* FTD *frontotemporal dementia,* FTLD *frontotemporal lobar degeneration,* FUS *fused in sarcoma protein,* MRI *magnetic resonance imaging,* MSA *multiple system atrophy,* NGS n*ext-generation sequencing,* NfL *neurofilament light chain, *p-tau181/217 *phosphorylated tau at threonine 181/217,* PET *positron emission tomography,* RBD *rapid eye movement sleep behavior disorder, *SOD1 *superoxide dismutase 1 protein/gene,* TDP-43 *transactive response DNA-binding protein of 43 kDa

In other neurodegenerative diseases – including synucleinopathies, frontotemporal lobar degeneration (FTLD), amyotrophic lateral sclerosis (ALS) and limbic-predominant age-related TDP-43 encephalopathy (LATE) – fluid biomarkers development remains challenging because of clinical heterogeneity and the absence of robust markers for hallmark pathologies. Reliable fluid biomarkers for synucleinopathies have long been sought. These disorders include Parkinson's disease (PD), the second most common neurodegenerative disease, characterized by intraneuronal α-synuclein inclusions; dementia with Lewy bodies (DLB), which also shows intraneuronal α-synuclein inclusions, frequently associated with AD co-pathology [7, 8]; and multiple system atrophy (MSA), where α-synuclein aggregates primarily in oligodendrocytes.

FTLD is another relevant cause of dementia – particularly early onset dementia – and is notable for its high heritability [9]. FTLD encompasses a heterogeneous spectrum of pathologies, mainly transactive response DNA-binding protein 43 (TDP-43) and tau proteinopathies [10], and less commonly FET proteinopathies. This pathological heterogeneity, together with the lack of large cohorts of pathologically confirmed cases, has hindered biomarker development. FTLD-TDP overlaps with ALS, a rare rapidly progressive neurodegenerative disease characterized by motor neuron degeneration [11]. ALS is most associated with TDP-43 pathology, although in some cases protein aggregates of mutated gene products may be found. Diagnosis relies on clinical, neuroimaging and electrophysiological assessment, as biomarkers are not yet available [12]. Notably, TDP-43 proteinopathy also occurs in LATE a late-onset condition clinically resembling AD, for which no established antemortem biomarkers are currently available.

**Fig. 1:**
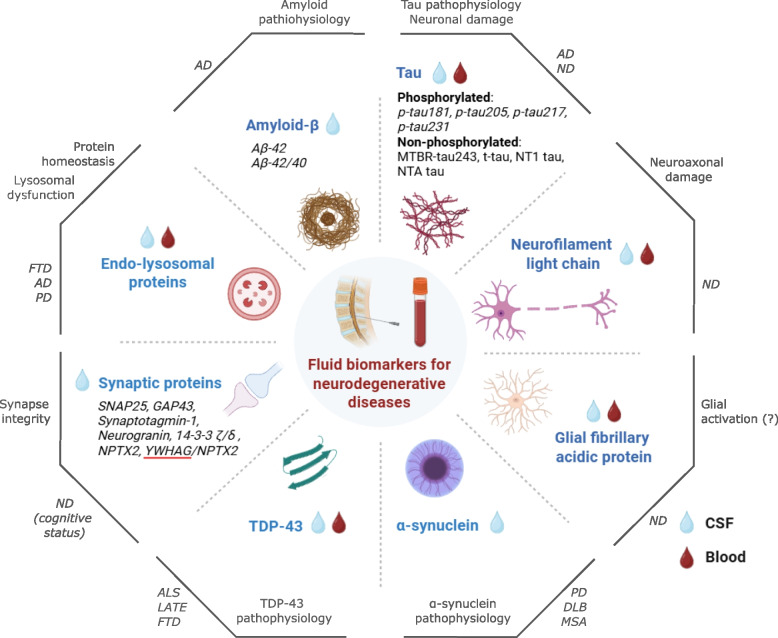
Overview of fluid biomarkers for neurodegenerative diseases covered in the review. Biomarker (groups) are assigned a biological process they are considered to reflect (normal font, gray), and associated diseases (italic font, gray). Abbreviations: Aβ: amyloid-beta; AD: Alzheimer's disease; ALS: amyotrophic lateral sclerosis; CSF: cerebrospinal fluid; DLB: dementia with Lewy bodies; GAP-43: growth associated protein-43 kDa; FTD: frontotemporal dementia; LATE: limbic-predominant age-related TDP-43 encephalopathy; MSA: multiple system atrophy; MTBR-tau243; microtubule-binding region tau 243; ND: neurodegenerative disease; NPTX2: neuronal pentraxin-2; NTA-tau: N-terminal containing tau fragments; PD: Parkinson’s disease; p-tau: phosphorylated tau; SNAP-25: synaptosomal-associated protein 25; TDP-43: transactive response DNA-binding protein of 43 kDa.; t-tau: total tau

In this review, we aim to provide a comprehensive overview of fluid biomarkers for neurodegenerative diseases, with a primary focus on AD and other common neurodegenerative disorders (Table 1). We summarize the state of the art and recent methodological advances, covering both well-established biomarkers such as amyloid-β and tau, as well as emerging candidates including synaptic and endo-lysosomal biomarkers (Fig. 1). Finally, we discuss the major challenges that remain for biomarker validation and clinical implementation. The methodology used for the literature review is described in the Supplementary Material.

### Current state of fluid biomarkers

#### Amyloid-β

Aβ is a small peptide generated by the enzymatic cleavage of amyloid precursor protein by β- and γ-secretases. This process produces Aβ peptides of varying lengths, with Aβ42 being particularly prone to aggregation [13–17]. Under normal conditions, Aβ is produced and cleared efficiently [18]. However, in AD, this balance is disrupted, leading to the accumulation of Aβ in extracellular plaques. Although the mechanisms of Aβ-induced neurodegeneration are not fully understood, soluble Aβ oligomers are believed to play a key role by disrupting synapses, injuring neurites, triggering oxidative stress and glial reactivity, ultimately contributing to neuronal loss [4, 19].

FDA-approved assays for CSF Aβ42 and Aβ40 have long been used in clinical settings, offering a reliable biomarker framework for the early diagnosis [5]. In AD, CSF Aβ42 levels are markedly reduced, reflecting its sequestration into amyloid plaques in the brain. Aβ40 levels remain relatively stable, serving as a normalization factor to control for individual differences in amyloid production and sampling variability. Consequently, the Aβ42/Aβ40 ratio has emerged as a more robust and specific diagnostic marker than Aβ42 alone [20, 21].

Aβ42 and Aβ40 can also be measured in blood, providing a less invasive alternative to CSF sampling for assessing amyloid pathology [22, 23]. Among the available analytical methods, mass spectrometry-based techniques (Table 3) are currently the most sensitive and accurate for quantifying Aβ peptides in plasma. However, plasma Aβ concentrations show a weaker and more variable correlation with brain amyloid deposition compared to CSF biomarkers [24]. The relatively small differences in plasma Aβ42 and Aβ40 levels between AD patients and controls, combined with variability that limits robustness, pose challenges for accurate amyloid detection [25–27]. Circulating Aβ levels are particularly sensitive to pre-analytical sample handling and to peripheral factors, including protein binding, renal clearance, and platelet-derived Aβ. These factors hinder the broader adoption of plasma Aβ assays in routine clinical practice.

#### Tau

Tau is a microtubule-associated protein encoded by the *MAPT* gene on chromosome 17. In the adult human brain, alternative splicing of MAPT gives rise to six tau isoforms, which differ in the number of N-terminal inserts and microtubule-binding repeats [28]. Tau plays a vital role in maintaining neuronal architecture and supporting axonal transport by stabilising microtubules and it also contributes to synaptic function**.** Under physiological conditions, tau remains a soluble, non-aggregating protein [29]. However, in AD and other tauopathies, tau becomes dysregulated, detaching from microtubules and aggregating into insoluble structures [30, 31]. This aggregation follows a progressive sequence from monomers to oligomers, ultimately forming paired helical filaments – a pathological hallmark of tauopathies. Aberrantly modified tau species, particularly hyperphosphorylated or aggregated forms, are thought to exert neurotoxic effects by destabilizing microtubules, impairing intracellular transport, and contributing to synaptic dysfunction [32].

##### Phosphorylated tau biomarkers in AD

The revised Alzheimer's Association criteria for the diagnosis and staging of AD [5] categorize tau biomarkers into two groups: T1 biomarkers, which reflect early tau hyperphosphorylation associated with Aβ pathology, and T2 biomarkers, which indicate later-stage tau aggregation and neurofibrillary tangle formation.

T1 biomarkers include several phosphorylated tau species such as p-tau181, p-tau217, and p-tau231 [33]. CSF p-tau181 was the first to be validated and correlates well with both Aβ and tau pathology [34]. While CSF p-tau181 remains widely used in routine practice, technological advances have enabled sensitive detection of p-tau181 and other p-tau species in blood [35–38]. Among these, plasma p-tau217 has emerged as the most robust BBM for AD [39]. It increases in Aβ PET-positive individuals, correlates with cognitive decline and brain atrophy [40–44], and shows diagnostic performance comparable or superior to FDA-approved CSF assays [45].

Other p-tau isoforms offer stage-specific information. Plasma p-tau231 rises in the earliest disease stages, even before Aβ PET positivity [46–48]. Conversely, p-tau205 is more closely linked to advanced tau aggregation and neurofibrillary tangle burden and is thus considered as a T2 marker [49]. A recent mass spectrometry and autopsy study confirmed that p-tau231, p-tau217, and p-tau205 track distinct phases of AD progression, reinforcing the utility of a multi-marker strategy for disease staging [50].

P-tau biomarkers are also instrumental for differential diagnosis. CSF p-tau181 is consistently elevated in AD but remains within normal ranges in non-AD tauopathies [51]. Likewise, plasma p-tau217 and p-tau231 have shown strong discriminatory power in differentiating AD from other neurodegenerative diseases [47]. Nevertheless, a recent study found elevated levels of p-tau181 and p-tau217 in serum and muscle of patients with ALS [52] and elevated CSF levels of p-tau isoforms have been observed in Creutzfeldt Jakob disease (CJD), particularly (but not only) in the presence of AD co-pathology [53].

More recently, phosphorylation at tau residues serine-262 and serine-356 have been proposed as a signature of early tau aggregation. Located within a soluble tau assembly core region, these sites selectively label granular, prefibrillar tau structures, distinguishing them from conventional p-tau markers [54]. A novel CSF assay targeting these epitopes effectively separated AD from non-AD tauopathies and correlated with tau PET, Braak stage, and cognitive status, independent of Aβ pathology [54].

##### Non-phosphorylated tau biomarkers in AD

Among the most promising non-phosphorylated tau biomarkers are tau fragments from the microtubule-binding region (MTBR) and non-phosphorylated N-terminal or mid-region tau species. CSF and plasma MTBR-tau243 have demonstrated potential value in identifying tau aggregation in AD [55, 56]. In one recent paper plasma MTBR-tau243 showed strong concordance with tau-PET and outperformed plasma %p-tau217 and %p-tau205 in capturing fibrillar tau pathology [56], which supports its utility as markers of insoluble tau burden pending independent replication studies.

Plasma N-terminal containing tau fragments (NTA-tau) captures tau species linked to neurofibrillary tangle pathology in AD [57]. NTA-tau correlates strongly with tau-PET but barely with Aβ biomarkers. Elevated NTA-tau levels are linked to advanced disease stages and predict tau accumulation, structural brain changes, and clinical worsening more effectively than plasma p-tau181 or total tau (t-tau) [57].

T-tau, while widely used in CSF, lacks specificity in blood due to substantial dilution effects from peripheral sources such as muscle and liver [31]. To overcome this, brain-derived tau (BD-tau) captures brain-specific tau isoforms, correlating more closely with structural brain changes and clinical status [58].

Despite the strong agreement between MTBR-tau243, NTA-tau, and BD-tau with tau-PET imaging and their superior performance compared to established tau markers, their development is still at an early stage with several limitations that persist. In particular, how these soluble tau biomarkers relate pathophysiologically to aggregated tau in the brain remains incompletely understood. Moreover, given that they appear to be indicators of later stages, this limits their utility for early detection and intervention.

##### Tau biomarkers in other neurodegenerative diseases

Tau pathology is also a defining feature of other neurodegenerative diseases [59], collectively referred to as tauopathies. Among the main tauopathies are progressive supranuclear palsy, corticobasal degeneration, and FTLD-tau, which includes Pick’s disease—a specific form characterized by 3R tau-positive Pick bodies, as well as genetic variants caused by *MAPT* mutations [29]. Tau aggregates also appear as co-pathology in ALS, Huntington’s disease, Niemann-Pick type C, and others [60]. Understanding isoform-specific tau patterns across diseases is essential for differential diagnosis.

CSF t-tau levels serve as a marker of neuronal injury and are elevated in AD as well as in other neurodegenerative diseases and in traumatic brain injury. While t-tau can help identify CJD, wherein its CSF levels are much higher than in other neurodegenerative diseases [61], low sensitivity and specificity at pathological intermediate levels (i.e. < 1200 pg/mL) limits its utility as a standalone diagnostic marker.

The pathological aggregates in tauopathies such as progressive supranuclear palsy and corticobasal degeneration are composed primarily of 4-repeat (4R) tau isoforms, whereas those in Pick's disease consist of 3-repeat (3R) tau isoforms [62]. Recent advances using immunoassays targeting extracellular vescicles (EVs) from plasma have enabled differentiation between 3 and 4R tau isoforms. For example, the EV 3R/4R tau ratio, especially when combined with EV TDP-43, shows promise for distinguishing FTLD-tau from FTLD-TDP [63]. In parallel, seed amplification assays (SAA) have demonstrated potential for detecting 3R and 4R tau isoforms with high sensitivity in FTLD-tau cases [64].

### Neurofilament light chain

Neurofilament light chain (NfL) is a cytoskeletal protein expressed in large caliber myelinated axons. Following axonal injury, NfL is released into the extracellular space, diffusing into the CSF and in the bloodstream. It is thus a marker of axonal injury, irrespective of the underlying cause [65]. The development of ultra-sensitive assays has significantly improved NfL measurement in blood [65]. Several studies consistently demonstrate higher NfL levels in patients with neurodegenerative diseases compared to controls, with highest levels found in CJD, ALS, FTLD and MSA, reflecting the aggressive nature of these conditions [66–68]. NfL concentrations also correlate strongly with severity and progression of various neurodegenerative diseases [69]. It is important to note that NfL levels in both CSF and blood naturally increase with age in healthy individuals, particularly after age 60 [70]. Currently, NfL is used to aid in the diagnosis of neurodegenerative diseases; however, as of its nonspecific nature, it can hardly distinguish between different disease entities. Another promising clinical application of NfL is predicting symptom onset in familial variants of neurodegenerative diseases. For example, in ALS, FTLD and Huntington’s disease higher levels have been reported in mutation carriers nearing symptom onset [71–73]. Additionally, NfL is used to evaluate treatment effects in clinical trials, for example in AD, as a reduction in NfL levels indicates a slower neurodegeneration, although p-tau217 and glial fibrillary acidic protein (GFAP) are suited better for monitoring purposes [74].

### Glial fibrillary acidic protein

GFAP is the major cytoskeletal constituent of astrocyte branches and a marker of astrocytic reactivity. In AD, reactive astrogliosis is an early response to Aβ and tau accumulation and can in turn contribute to the progression of the pathology [75, 76]. Therefore, GFAP was included in the revised Alzheimer’s Association criteria as a marker of inflammation [5]. A meta-analysis showed that GFAP can identify AD cases with positive PET or CSF biomarkers with an area under the curve (AUC) of 0.75 and 0.77, respectively [77]. Notably, blood GFAP performs even better than CSF GFAP in the identification of AD cases [77]. The reasons for this superiority remain unexplained, but it could be due to an excess release via astrocytic endfeet into the bloodstream, or to a lower stability in CSF [78]. Moreover, longitudinal studies demonstrated an association between elevated blood levels of GFAP and different indicators of disease progression, including accelerated brain atrophy, faster cognitive decline [79] and dementia onset [44, 79–82]. In individuals with Down syndrome, GFAP was shown to discriminate prodromal AD and AD cases from asymptomatic individuals [83]. GFAP is also elevated in other neurodegenerative and non-neurodegenerative conditions [84], emphasizing that reactive astrogliosis is a response to non-specific stimuli. Previous studies observed high blood levels of GFAP in individuals with FTLD or ALS, though not to the extent as seen in AD [85], and in those with DLB [86] and prion diseases [87].

### α-Synuclein

Recent methodological advancements, drawing inspiration from prion research, have led to the development of SAAs for detecting α-synuclein in CSF [88]. These assays show good correlation with Lewy body pathology confirmed at autopsy [89], can distinguish between individuals with PD and healthy controls with high sensitivity and specificity [90] and support differentiation between PD and other neurodegenerative diseases [91]. SAAs exhibit similarly robust sensitivity (92–93%) and specificity (96–100%) in identifying pathological α-synuclein in patients with DLB [92]. Observed differences in seeding activities between DLB and MSA may be attributable to distinct quaternary structures of protein aggregates [93]. Ongoing research is focused on enhancing the capacity of SAAs to discriminate between various synucleinopathies [94] and to map progression of Lewy body pathology [95]. Emerging evidence also indicates that SAAs could potentially be used to detect pathological α-synuclein in the serum of patients with synucleinopathies [96].

Specific α-synuclein species, such as oligomeric forms or genetically aggregation-prone phosphorylated serine 129 α-synuclein (pS129-aSyn), exhibit higher specificity for MSA [97]. Furthermore, the membrane-bound fraction of erythrocyte α-synuclein has been proposed as a potential biomarker for MSA [98].

Circulating plasma EVs contain elevated levels of α-synuclein in both DLB and MSA patients [93], but several challenges persist. These include standardizing isolation techniques, minimizing contaminants, and determining the specific cellular origins of EVs [99, 100].

### TDP-43 and related biomarkers

TDP-43 is integral to RNA metabolism; however, its pathological aggregation is a hallmark feature across a spectrum of neurodegenerative diseases, including ALS, FTLD-TDP and LATE. Efforts are underway to improve the detection of pathological TDP-43 in diverse biofluids.

SAA-based methods are currently under development for TDP-43 detection in CSF and show promise [101]. Nevertheless, relatively high false-positive rates limit their clinical applicability [102]. The diagnostic and prognostic utility of CSF and blood TDP-43 levels has been extensively investigated in ALS and FTD. However, studies have yielded inconsistent results regarding diagnostic accuracy, association with genetic variants, and correlation with disease progression [103–108]. Elevated blood TDP-43 levels have also been reported in AD, potentially indicating a co-pathology associated with an unfavourable prognosis [103].

A significant gap persists in the availability of fluid biomarkers for LATE. Indeed, the absence of reliable antemortem detection methods for its hallmark TDP-43 pathology not only limits the development of specific biomarkers [109], but also hampers accurate in vivo diagnosis and makes it difficult to distinguish LATE from other age-related dementias. One study reported an association between elevated plasma TDP-43 levels and brain atrophy in clinically suspected LATE cases [110]. Emerging biomarker candidates include cryptic exon-encoded peptides, such as those derived from HDGFL2, which serve as an indicator of dysfunctional TDP-43-mediated splicing [111], and TDP-43 protein in plasma brain-derived EVs [63] and phosphorylated TDP-43 in plasma [112].

### Synaptic proteins

Synaptic dysfunction is a recognized hallmark and functional correlate of neurodegenerative diseases. Disruption of presynaptic and postsynaptic compartments can lead to the release of synaptic proteins into the extracellular space, enabling their detection in CSF. However, to date the utility of these proteins as plasma biomarkers has not been established (Table 2) [113].

**Table 2:** Comparative detection status of synaptic protein biomarkers in CSF and plasma

SYNAPTIC PROTEIN	CSF DETECTION	PLASMA DETECTION
SNAP-25	Established	Not established
GAP-43	Established	Not established
SYNAPTOTAGMIN-1	Established	Not established
NEUROGRANIN	Established	Not established
14–3-3 ZETA/DELTA	Established	Limited data
AP2B1	Established	Limited data
SYNTAXIN-1B	Established	Limited data
NPTX2	Established	Limited data
NPTX1	Established	Limited data

Established detection indicates validated measurement with clinical utility demonstrated in published studies. Limited data refers to insufficient evidence for clinical application

Synaptosomal-associated protein 25 (SNAP-25), a presynaptic protein crucial for vesicular exocytosis, is emerging as a potential biomarker of synaptic degeneration [114]. SNAP-25 is reduced in the brain but increased in the CSF of AD patients [115–117]. CSF SNAP-25 levels can distinguish AD cases from controls with high accuracy (AUC 0.89) and were strongly associated with amyloid-PET, tau-PET, and cortical thickness [118]. Notably, SNAP-25 emerged as the strongest predictor of progression to AD dementia in non-demented individuals when compared other synaptic markers. Importantly, elevated CSF levels of SNAP-25 have also been reported in CJD [117, 119]. One study reported reduced CSF SNAP-25 levels in DLB, whereas levels were increased in DLB with AD co-pathology, suggesting its potential utility in differentiating pure DLB from DLB-AD [120].

Growth associated protein-43 kDa (GAP-43), a presynaptic protein predominantly expressed in mesiotemporal areas, was elevated in AD patients compared to controls and other neurodegenerative diseases. Its levels were already increased in preclinical AD [121] and were associated with higher CSF p-tau and t-tau, Aβ deposition, hippocampal atrophy, and worse cognitive performance [121–123].

Synaptotagmin-1, a presynaptic transmembrane protein essential for hippocampal neurotransmitter release, exhibits significantly increased CSF levels in patients with AD dementia and even mild cognitive impairment due to AD or preclinical AD compared to controls [124, 125].

Neurogranin, a postsynaptic marker involved in spatial memory, is consistently elevated in CSF in AD cases compared to controls [126–128] and has been linked to accelerated cognitive decline and hippocampal atrophy [129]. Notably, except for CJD, neurogranin elevation appears specific to AD.

Within the AD continuum, CSF 14–3-3 zeta/delta protein (ζ/δ) levels rise in subjects with Aβ deposition and early tau pathology, correlating primarily with neurodegeneration and memory function in cognitively unimpaired individuals. This observation further supports the premise that synaptic dysfunction is detectable in preclinical AD [130]. While 14–3-3 levels are also increased in FTLD patients, they are at levels intermediate between AD and PD/DLB patients [131].

Other proteins, such as adaptor related protein complex 2 subunit beta 1 (AP2B1) or syntaxin-1B have demonstrated lower CSF levels in FTLD compared to AD and controls [131]. Neuronal pentraxins are generally decreased in neurodegenerative diseases. Specifically, neuronal pentraxin-2 (NPTX2) is decreased in AD, FTD and LBD compared to controls, and neuronal pentraxin-1 (NPTX1) is decreased in AD and FTD patients but not in DLB [131]. A recent study has demonstrated that the CSF YWHAG/NPTX2 ratio predicts cognitive decline in AD [132].

### Endo-lysosomal biomarkers

Endo-lysosomal proteins represent a critical aspect of neurodegeneration, reflecting cellular protein homeostasis and lysosomal dysfunction. A targeted mass spectrometry study investigating AD and PD revealed significantly altered concentrations of proteins such as cathepsin B, cathepsin F, GM2 activator protein (GM2A), lysosome-associated membrane protein 2, AP2B1, and ubiquitin in the CSF of PD patients [133]. These findings suggest a generalized lysosomal dysfunction in PD, consistent with genetic insights. In contrast, no significant differences in peptide concentrations were observed between AD patients and controls, indicating less pronounced lysosomal involvement in AD compared to PD. In the blood, however, progranulin levels are reported to be increased in AD and mild cognitive impairment [134].

In a spectrum of clinical aphasia syndromes associated with FTD/FTLD, alterations in CSF profiles of endo-lysosomal proteins, as measured by targeted mass spectrometry, could differentiate most subtypes from healthy controls [135]. Specifically, in the non-fluent variant of primary progressive aphasia, multiple cathepsins, AP2β, GM2A, ubiquitin and other proteins were found to be decreased. The semantic variant exhibited a distinct protein profile, with reductions in a different set of proteins including Aβ A4, cathepsin Z, and dipeptidyl peptidase 2. Interestingly, in the logopenic variant, which frequently features AD pathophysiology, no significant protein alterations were identified. Another study on genetic FTLD caused by mutation in the *CHMP2B* gene, coding for charged multivesicular body protein 2b, revealed higher CSF levels of complement component 9, lysozyme, and transcobalamin 2 compared to family controls, alongside reduced ubiquitin, CTSB, and amyloid precursor protein levels [136].

### Clinical-neuropathological correlation and validity of fluid biomarkers

Clinical and neuropathological diagnoses do not always align perfectly. Indeed, a substantial portion of clinically diagnosed cases of AD and other neurodegenerative diseases are not confirmed at autopsy [137]. Neuropathologically validated cohorts are indispensable for establishing the diagnostic accuracy of fluid biomarkers, as neuropathological examination remains the gold standard for diagnosis in sporadic neurodegenerative diseases. In AD, CSF biomarkers (p-tau181, Aβ42) and their ratios have a good accuracy for identifying AD neuropathologic change with AUC values around 0.95–0.96 [138]. With recent progress in BBMs, similar performances have been obtained by plasma p-tau217, ptau-181 and ptau-212 [50, 139–141]. In one study involving 142 autopsy-proven AD, FTLD/ALS and PD/DLB cases, the clinical-neuropathological diagnostic concordance was 81.4% [142] and reliance on clinical rather than neuropathological diagnoses led to an underestimation of CSF biomarkers accuracy by 14–17% [142]. The presence of concomitant neuropathologies further complicates fluid biomarkers validation and accurate antemortem diagnosis. For example, abnormal AD biomarkers also detect AD co-pathology in cases with non-AD primary diagnoses [143]. Conceptually, fluid biomarkers can also be viewed from a more disease-agnostic modular perspective by focusing on their correlation with isolated neuropathological traits.

### Technological advances in fluid biomarker detection

Recent innovations in fluid biomarker detection technologies have significantly enhanced analytical sensitivity, specificity, and throughput. These advancements are transforming biomarker measurement from a research-intensive endeavor into a scalable clinical possibility [37, 144, 145].

Mass spectrometry is widely used for comprehensive proteomic profiling and for accurate quantification of disease-related proteins with the potential for reference-quality measurements. Through precise mass determination of analyte molecules, thousands of proteins in CSF or plasma can be identified and relatively quantified. Mass spectrometry also enables characterization of posttranslational modifications and proteolytic processing. Advances in instrumentation and analytical workflows have made mass spectrometry-based proteomics feasible in large-scale clinical studies. Unlike immunoassays, which are limited to predefined targets, mass spectrometry-based approaches provide an unbiased, hypothesis-generating platform for protein biomarker discovery, making the two approaches complementary. For validation and further development of new biomarker candidates, mass spectrometry is often the method of choice as targeted assays can be developed quickly, often without the need for antibodies, and because the technique allows parallel measurement of multiple proteins in a single analysis. In AD research, mass spectrometry has been used to quantify plasma biomarkers such as p-tau, Aβ peptides, and NfL [37, 45, 48, 49, 146, 147]. While label-free mass spectrometry offers broad, untargeted proteomic coverage, targeted strategies like multiple reaction monitoring (MRM) enable high-sensitivity detection of specific proteins.

Single molecule array (Simoa) is a bead-based digital immunoassay platform capable of quantifying proteins at femtomolar concentrations by isolating and digitally counting individual immunocomplexes. It has become a cornerstone for fluid biomarker quantification in AD, particularly for Aβ, p-tau isoforms, NfL, and GFAP. Recent developments have further enhanced assay specificity for p-tau217 and GFAP, improving differentiation between AD and other neurodegenerative disorders [148, 149].

The proximity extension assay (PEA), developed by Olink, utilizes DNA-tagged antibody pairs and nucleic acid amplification for high-throughput protein quantification, allowing the measurement of over 3,000 proteins from low-volume samples. As of 2025, the latest PEA panels have identified inflammation and synapse-related profiles that are predictive of preclinical AD progression, and these are currently being evaluated for trial enrichment and patient stratification [150].

Nucleic acid linked immuno-sandwich assay (NULISA) combines the specificity of immunoassays with nucleic acid amplification to detect a broad panel of proteins from the central nervous system in plasma. The updated NULISA-Seq CNS Panel 2.0, released in 2025, demonstrated improved dynamic range and detection limits for p-tau181, NfL, YKL-40, and neurogranin. Validation studies have shown strong concordance with CSF A/T/N profiles, positioning NULISA as a competitive alternative to Simoa [112, 151].

Automated immunoassay platforms, including Roche Elecsys and Fujirebio Lumipulse, are already in clinical use for CSF biomarkers and are rapidly being adapted for plasma. In 2025, the Elecsys platform incorporated plasma p-tau217, enabling high-throughput, standardized measurement [27]. SAAs, including Real-Time Quaking-Induced Conversion (RT-QuIC) and Protein Misfolding Cyclic Amplification (PMCA), are ultrasensitive techniques that amplify misfolded protein “seeds” to detect pathological tau and α-synuclein in CSF [152]. Originally developed for prion diseases, these assays have been successfully adapted for other neurodegenerative disorders.

Collectively, these platforms (summarized in Table 3) form an integrated and maturing toolkit for diagnostics, marking a shift toward scalable, precise, and early biomarker-based diagnosis in neurodegenerative diseases.

**Table 3:** Comparison of technologies for fluid biomarker discovery and validation

	IMMUNOASSAYS									
CATEGORY	Explorative					Targeted				
METHOD	PEA	Olink	NULISA	Luminex		SIMOA		CLIA	ELLA	ELISA
PRINCIPLE	Dual antibodies linked to oligonucleotides	Advanced PEA with proprietary technology	Links nucleic acids to immunoassays	Multiplex bead-based immunoassay		Digital ultra-sensitive immunoassay		Enzyme-linked chemiluminescent reaction for antigen detection	Automated multiplex microfluidic immunoassay	Antibody-based antigen detection
SENSITIVITY	Very High	Very High	Ultra-High	High		Ultra-High		Very High	High	Moderate
SPECIFICITY	High	High	High	High		High		High	High	High
THROUGHPUT	High	High	High	High		Moderate		High	Moderate	Moderate
SAMPLE TYPE	CSF, Blood	CSF, Blood	CSF, Blood	CSF, Blood		CSF, Blood		CSF, Blood	CSF, Blood	CSF, Blood
KEY BIOMARKERS	Aβ42/40, p-tau181, NfL, GFAP, inflammatory markers, synaptic dysfunction, Tau isoforms	Aβ42/40, p-tau181, NfL, GFAP, Tau, inflammatory markers	Aβ42/40, p-tau181, p-tau217, Tau, NfL, GFAP	Aβ42/40, p-tau181, NfL, GFAP, cytokines, chemokines, growth factors		Aβ42/40, p-tau181, p-tau217, NfL, GFAP, Tau		Aβ42/40, total tau, p-tau181	Aβ42/40, p-tau181, NfL, GFAP	Aβ42/40, total tau, p-tau181, p-tau217, NfL, GFAP
MULTIPLEX CAPABILITY	High	High	High	High		Intermediate		Low	Low	Low
COST	Expensive	Expensive	Expensive	Expensive		Expensive		Affordable	Expensive	Affordable
APPLICATIONS	Research, Discovery	Research, Discovery	Research, Discovery	Research, Clinical		Research, Clinical		Clinical, Research	Research, Clinical	Research, Clinical
DATA ANALYSIS	Moderate	Moderate	Complicated	Moderate		Moderate		Easy	Easy	Easy
ADVANTAGES	High multiplexing; small sample requirements; robust quantification; scalable for discovery studies	Minimal sample volume; detects > 3000 proteins; excellent for large-scale studies	Surpasses SIMOA in sensitivity; supports quantitative biomarker detection	Flexible; detects multiple biomarkers in one sample; high reproducibility		First and best-validated ultrasensitive technology for targeted low-plex biomarker measurements		Automated; high throughput; highly sensitive for routine testing	Fast and automated; high precision; minimal hands-on time	Gold-standard for clinical diagnostics; inexpensive
LIMITATIONS	Requires antibodies; provides only relative quantification	Proprietary reagents; relative quantification only	Emerging technology; requires validation and clinical adoption	Requires dedicated instruments; lower sensitivity compared to single-plex methods		Limited multiplexing; instrument availability restrictions		Limited multiplexing; dependency on antibody specificity	Lower multiplexing compared to MSD or PEA	Limited sensitivity for low-abundance proteins; single-analyte focus
	Mass spectrometry assays								Seed Amplification Assays	
CATEGORY	Explorative		Targeted						Explorative	
METHOD	Label-Free LC–MS		PRM-MS		SRM-MS		IP-MS		RT-QuIC/PMCA	
PRINCIPLE	Measures mass-to-charge ratio of peptides/proteins		Monitors all product ions from a precursor ion		Monitors transitions from precursor to product ions		Combines antibody enrichment with MS		Detects and amplifies pathological misfolded seeding-competent protein	
SENSITIVITY	High		Very High		High		High		Ultra-High	
SPECIFICITY	High		Very High		High		High		Very High	
THROUGHPUT	Low		Moderate		Moderate		Low		Moderate	
SAMPLE TYPE	CSF, Blood		CSF, Blood		CSF, Blood		CSF, Blood		CSF, Blood, nasal swab, skin	
KEY BIOMARKERS	Aβ42/40, p-tau181, p-tau217, Tau, ApoE isoforms		Aβ42/40, p-tau181, Tau isoforms, NfL		Aβ42/40, p-tau181, Tau isoforms, NfL		Aβ42/40, p-tau181, p-tau217, SNAP-25		α-synuclein, tau, prion protein	
MULTIPLEX CAPABILITY	Low		Intermediate		Intermediate		Low		Low	
COST	Expensive		Expensive		Expensive		More Expensive		Affordable-Expensive	
APPLICATIONS	Research, Discovery		Research, Clinical		Research, Clinical		Research, Targeted		Research, Clinical	
DATA ANALYSIS	Complicated		Complicated		Complicated		Complicated		Moderate	
ADVANTAGES	High specificity; antibody-independent; ideal for PTM and discovery studies		Quantitative and isoform-specific analysis		Robust quantitative capabilities		Sensitivity for low-abundance proteins; excellent for PTM characterization		Detects pathological protein aggregates with high sensitivity, functional readout	
LIMITATIONS	Complex workflows; requires expertise		Requires optimization		Predefined targets limit discovery		Complex sample preparation workflows		Requires thorough protocol optimisation, specialized equipment and reagents	

*Abbreviations ApoE *apolipoprotein E,* CLIA *chemiluminescence immunoassay,* CSF *cerebrospinal fluid,* ELLA *enzyme-linked lectin assay (ProteinSimple/Bio-Techne),* ELISA *enzyme-linked immunosorbent assay,* GFAP *glial fibrillary acidic protein,* IP-MS *immunoprecipitation-mass spectrometry,* LC–MS *liquid chromatography-mass spectrometry,* NfL *neurofilament light chain,* NULISA *nucleic acid linked immunosorbent assay,* PEA *proximity extension assay,* PRM-MS *parallel reaction monitoring-mass spectrometry,* PTM *post-translational modification,* RT-QuIC *real-time quaking-induced conversion,* SIMOA s*ingle-molecule array,* SNAP-25 *synaptosomal-associated protein 25,* SRM-MS *selected reaction monitoring-mass spectrometry

### Clinical integration and applicability of blood-based biomarkers

While the development of first CSF-based biomarkers from discovery to final regulatory approval has spanned over two decades, the current advances for BBMs implementation benefit partly from accelerated processes.

**Fig. 2:**
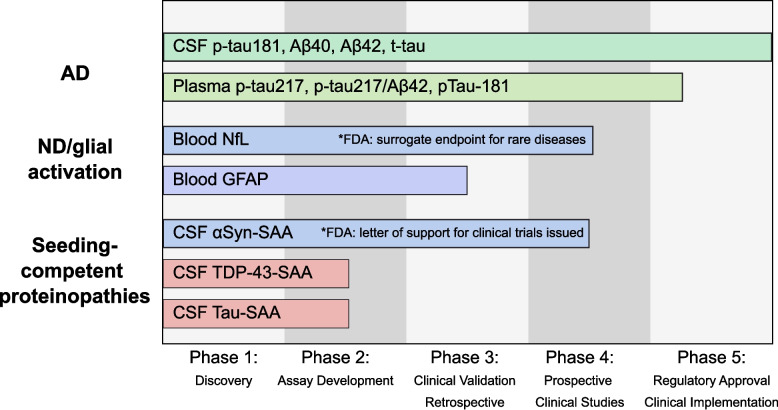
Developmental and regulatory status of selected CSF and blood biomarkers mapped to the 5-step strategic roadmap for validating biomarkers. Bars indicate the approximate phase reached: plasma p-tau217 and the p-tau217/Aβ42 ratio have FDA clearance for assessing amyloid pathology in symptomatic adults in the USA [169] (not EU); NfL is accepted as a reasonably likely surrogate endpoint in SOD1-ALS [170, 171]; and α-synuclein SAA has an FDA Letter of Support for use in clinical trials [172]. Status labels reflect evidence and regulatory positions as of 2025 and may evolve with ongoing standardization and validation efforts. Abbreviations: Aβ: amyloid-beta; AD: Alzheimer's disease; αSyn, α-synuclein; CSF: cerebrospinal fluid; GFAP, glial fibrillary acidic protein; ND: neurodegenerative disease; NfL, neurofilament light chain; p-tau: phosphorylated tau; SAA, seed-amplification assay; TDP-43: transactive response DNA-binding protein of 43 kDa.; t-tau: total tau

In 2017, an international working group outlined a 5-step strategic roadmap for validating AD biomarkers (Fig. 2) [153]. Phases 1–3, dedicated to proving clinical-analytical validity, are largely accomplished for most promising BBMs like p-tau217 and 181. Yet, challenges remain with real-world applicability (phases 4–5) for these AD markers. More extensive assay development and clinical validation (phases 2–4) are also needed for broader biomarkers such as NfL, GFAP, and markers of seeding-competent proteinopathies like TDP-43 and tau (Fig. 2) [154]. Pre-analytical factors such as specimen collection, processing times, storage conditions, and assay sensitivity limitations should meticulously be accounted for [155]. As conceptual derivates from established CSF biomarkers, one major challenge with BBMs is the significant concentration gradient of brain-derived biomolecules in blood, a technically relevant limitation that has been partly mitigated by recent ultrasensitive quantitation techniques [151, 156, 157]. However, variability in BBM levels due to sociodemographic factors (e.g., age, ethnicity, and sex), comorbidities (e.g. kidney dysfunction) and medications (e.g., sacubitril/valsartan) [158–162] remain significant obstacles, particularly given their use in aged multimorbid populations. Ethnic differences may influence biomarker levels [163, 164], though these effects are partly explained by social determinants of health and comorbidities [165], highlighting the need for more ethnically diverse cohorts and global collaborations to close these research gaps. Sex differences also impact biomarker profiles and clinical progression, particularly noted in AD and FTD [166, 167]. Applying BBM ratios may attenuate the influence of some confounders [168].

Cross‑marker and cross‑disease comparability remains limited due to the scarcity of meta‑analyses and substantial heterogeneity in reference standards (PET, CSF, clinical diagnosis, autopsy), assay platforms, and non‑predefined thresholds, all of which reduce the interpretability of pooled performance across studies and cohorts. As an exception, a recent large meta‑analysis (113 studies; n = 29,625) identified plasma p‑tau217 as the best performer for biologically defined AD (sensitivity 88.1%, specificity 88.7%, AUC 91.1%), yet ~ 90% of studies were high risk of bias from non‑predefined thresholds – underscoring the need for prospective implementation and standardized cut‑offs [39].

Successful clinical implementation of BBMs requires standardized cut-offs and clear understanding of their relationship with clinical outcomes. Ongoing efforts in biomarker standardization and harmonization aim to reduce inter-laboratory variability and improve assay comparability, enabling widespread clinical adoption [173]. A two-cutoffs model that categorizes patients as positive, intermediate, or negative has shown better accuracy in detecting amyloid pathology than binary (positive/negative) cut-offs [42, 174, 175]. Interpretation is made more difficult, though, by the limited capacity to forecast disease progression in BBM-positive individuals. Establishing positive and negative predictive values will help physicians interpret and convey findings, therefore guiding them and supporting clinical decision-making. This is particularly important for primary care physicians, who often serve as the initial contact and may require specific training for biomarker interpretation [175, 176] Ethical concerns also arise, as many BBM-positive patients won’t have access to disease-modifying therapies, which underscores the importance of carefully evaluating the clinical utility of testing.

Future studies should focus on enhancing prognostic accuracy with emerging biomarkers, clarifying their specific contexts of use and validating their predictive value across diverse populations and disease stages. Combining multiple biomarkers that capture different pathophysiological processes – such as amyloid deposition, tau pathology, synaptic dysfunction, and neuroinflammation – can improve diagnostic precision and better predict clinical progression, for example, by using the CSF YWHAG:NPTX2 ratio or a panel of plasma p-tau181, p-tau217, NfL, and GFAP [44, 132, 177]. Multiplex biomarker panels could also facilitate patient stratification, for instance, through the implementation of specific plasma tau species [178], and allow for individualized treatment approaches [179], which are essential for personalized medicine strategies in neurodegenerative diseases. Notably, most biomarker research to date has focused strongly on AD, while non-AD neurodegenerative diseases have received less attention, limiting diagnostic and therapeutic advances in these conditions.

### Readiness for clinical application

Regulatory approval of biomarkers is essential for harmonization of diagnostic workflows, integration into drug development pipelines, and cost coverage by health insurances. Through a cooperative process that starts with defining the biomarker's context of use and concludes with a qualification decision, the FDA's Biomarker Qualification Program in the US directs biomarker approval for research use. To speed up the approval of promising technologies the FDA also provides a Breakthrough Device Designation. The European Medicines Agency's "Qualification of Novel Methodologies" program in Europe promotes the regulatory alignment of biomarkers in drug development [180, 181]. In May 2025, Fujirebio’s plasma-based Lumipulse G assay for determining the p-tau217/Aβ42 ratio as proxy for amyloid plaque presence in AD is the first to be approved by the FDA (Fig. 2) [169]. In October 2025, the Elecsys p-tau181 plasma assay from Roche was approved by the FDA for use in primary care settings [182].

Regulatory decisions for disease-modifying treatments have been impacted by well-documented but still unapproved biomarkers. For example, aducanumab and lecanemab's FDA approval was supported by biomarker evidence of decreased Aβ and tau pathology, despite the fact that their phase 3 trials revealed inconsistent clinical results [183–185]. Likewise, the FDA and EMA approved Tofersen, an antisense medication for SOD1-ALS based on the reduction of plasma NfL and CSF SOD-1 levels [170, 171]. For α-synuclein SAA the FDA has recently issued a Letter of Support for use in clinical trials [172].

## Conclusions

Fluid biomarker research is rapidly transforming neurodegenerative disease diagnostics, enabling early detection and precision medicine approaches through individualized pathological profiling. However, progress in widespread clinical adoption remains hindered by the absence of standardized assays and harmonized reference values.. These challenges arise from both technical factors, including variable pre-analytical protocols, assay heterogeneity, and lack of certified reference materials. In addition biological factors, such as age-related changes and presence of co-pathologies should also be carefully studied to grant an accurate interpretation of the biomarker results.

Future efforts should prioritize: (1) studies assessing biomarker pathophysiology to improve interpretation of biomarker results, (2) development of harmonized workflows and reference standards, (3) multi-platform validation studies across diverse populations, (4) longitudinal investigations defining biomarker trajectories and clinical utility, and (5) comprehensive cost-effectiveness evaluations. Addressing these critical gaps will enable translation of early-stage biomarker candidates into reliable, accessible diagnostic tools that improve patient care in neurodegenerative diseases.

## Supplementary Information

Supplementary Material 1.

## Data Availability

No datasets were generated or analysed during the current study.

## Abbreviations

AA: Alzheimer’s Association
AD: Alzheimer’s Disease
ALS: Amyotrophic Lateral Sclerosis
Aβ: Amyloid-β
BBM: Blood-Based Biomarker
BD-tau: Brain-Derived Tau
CJD: Creutzfeldt-Jakob Disease
CSF: Cerebrospinal Fluid
DLB: Dementia with Lewy Bodies
EV: Extracellular Vesicle
FDA: Food and Drug Administration
FTLD: Frontotemporal Lobar Degeneration
FTLD-tau: Tau-Positive Frontotemporal Lobar Degeneration
FTLD-TDP: TDP-43-Positive Frontotemporal Lobar Degeneration
FUS: Fused in Sarcoma (gene/protein)
GAP-43: Growth Associated Protein-43 kDa
GFAP: Glial Fibrillary Acidic Protein
GM2A: GM2 Activator Protein
LATE: Limbic-Predominant Age-Related TDP-43 Encephalopathy
MAPT: Microtubule-Associated Protein Tau
MRM: Multiple Reaction Monitoring (mass spectrometry)
MSA: Multiple System Atrophy
MTBR: Microtubule-Binding Region (of tau)
NfL: Neurofilament Light Chain
NPTX1/2: Neuronal Pentraxin-1/-2
NTA-tau: N-Terminal Containing Tau Fragments
NULISA: Nucleic Acid Linked Immuno-Sandwich Assay
PEA: Proximity Extension Assay
PET: Positron Emission Tomography
PMCA: Protein Misfolding Cyclic Amplification
p-tau: Phosphorylated Tau
pS129-aSyn: Phosphorylated Serine-129 α-Synuclein
RT-QuIC: Real-Time Quaking-Induced Conversion
SAA: Seeding Amplification Assay
Simoa: Single Molecule Array
SNAP-25: Synaptosomal-Associated Protein-25
SV2A: Synaptic Vesicle Glycoprotein-2A
TDP-43: Transactive Response DNA-Binding Protein-43 kDa
t-tau: Total Tau
3R/4R tau: Three-Repeat/Four-Repeat Tau Isoforms
