# Supplementary material for: Fluid biomarkers for neurodegenerative diseases: a comprehensive update
Source: Alzheimers Res Ther. 2025 Dec 20;18:12. doi: 10.1186/s13195-025-01919-z (PMC12805704; doi:10.1186/s13195-025-01919-z)
Supplement: Supplementary file 1 — Supplementary Material 1. [file 13195_2025_1919_MOESM1_ESM.docx]

**Supplementary material**

**Supplementary methods**

We reviewed studies assessing fluid biomarkers (i.e., measured in cerebrospinal fluid [CSF] or blood) in neurodegenerative diseases. Literature searches were conducted in the PubMed, Web of Science, and Embase databases without restrictions on time or language. We used a combination of MeSH terms and free-text keywords referring to CSF biomarkers, blood biomarkers, amyloid-beta (Aβ), tau, neurofilament light chain (NfL), glial fibrillary acidic protein (GFAP), alpha-synuclein (α-synuclein), TAR DNA-binding protein 43 (TDP-43), synaptic proteins, endolysosomal biomarkers, and neurodegenerative diseases including Alzheimer’s disease (AD), Parkinson’s disease (PD), dementia with Lewy bodies (DLB), multiple system atrophy (MSA), synucleinopathies, frontotemporal lobar degeneration (FTLD), limbic-predominant age-related TDP-43 encephalopathy (LATE), and amyotrophic lateral sclerosis (ALS). References cited in selected papers and other relevant articles were screened for additional studies following a snowball approach. Data were synthesized according to the type of biomarker.

The literature review was conducted by M.V., N.B., I.Y., M.B., A.C., J.D., A.F., A.G.M., G.I., F.M.D., and S.M.
